# Mild anemia as a single independent predictor of mortality in patients with COVID‐19

**DOI:** 10.1002/jha2.167

**Published:** 2021-05-06

**Authors:** Douglas Tremblay, Joseph L. Rapp, Naomi Alpert, Wil Lieberman‐Cribbin, John Mascarenhas, Emanuela Taioli, Saghi Ghaffari

**Affiliations:** ^1^ Department of Medicine Division of Hematology Oncology Icahn School of Medicine at Mount Sinai New York New York USA; ^2^ Institute for Translational Epidemiology Icahn School of Medicine at Mount Sinai New York New York USA; ^3^ Tisch Cancer Institute Icahn School of Medicine at Mount Sinai New York New York USA; ^4^ Department of Cell, Developmental & Regenerative Biology Icahn School of Medicine at Mount Sinai New York New York USA

**Keywords:** anemia, COVID‐19, haemoglobin, mortality, prognosis, RBC

## Abstract

The coronavirus disease 2019 (COVID‐19) caused by Severe Acute Respiratory Syndrome Coronavirus 2 (SARS‐CoV‐2) has led to an unprecedented international health crisis. COVID‐19 clinical presentations cover a wide range from asymptomatic to severe illness and death. Given the limited therapeutic resources and unexpected clinical features of the disease, readily accessible predictive biomarkers are urgently needed to improve patient care and management. We asked the degree to which anemia may influence the outcome of patients with COVID‐19. To this end, we identified 3777 patients who were positively diagnosed with COVID‐19 between March 1 and April 1 2020 in New York City. We evaluated 2,562 patients with available red blood cell, hemoglobin, and related laboratory values. Multivariable cox proportional hazards regression showed that anemia was a significant independent predictor of mortality (hazard ratio (HR): 1.26, 95% Confidence Interval [CI]: 1.06‐1.51), independent of age, sex, and comorbidities. There was a direct correlation between the degree of anemia and the risk of mortality when hemoglobin was treated as a continuous variable (HR_adj_ 1.05; [CI]: 1.01‐1.09). The hemoglobin level that was maximally predictive of mortality, was 11.5 g/dL in males and 11.8 g/dL in females. These findings identify a routinely measured biomarker that is predictive of disease outcomes and will aid in refining clinical care algorithms and optimize resource allocation. Mechanisms of impacts of anemia on COVID‐19 outcome are likely to be multiple in nature and require further investigation.

## 1 INTRODUCTION

The global coronavirus disease 2019 (COVID‐19) is caused by Severe Acute Respiratory Syndrome Coronavirus 2 (SARS‐CoV‐2) and was initially observed in late 2019 in Wuhan (Hubei Province), China and has led to an extraordinary global health crisis. Worldwide efforts have rapidly pinpointed biomarkers of disease severity and mortality that are currently guiding patient management [1, 2]. SARS‐CoV‐2 primarily employs angiotensin‐converting enzyme‐2 (ACE‐2) [3, 4] expressed on a variety of cell types [5], to alter cellular homeostasis [6]. However, not all cells involved in the pathogenesis of the disease are directly infected by the virus. For instance, despite the lack of viral invasion, monocytes and macrophages are critical in influencing COVID‐19 disease severity by regulating cytokine production and stimulating adaptive immune response. Although many blood biomarkers including lymphopenia, inflammatory indices such as high levels of circulating pro‐inflammatory cytokines specifically inerleukin‐6 (IL‐6) and TNF‐α, ferritin and coagulation factors have been reported to be associated with severe COVID‐19 [1], their predictive value for the disease course and severity is not yet fully established [7, 8].

While our understanding of the pathophysiology of the disease is rapidly evolving, additional indices that might identify patients who are most at risk of severe outcome in order to inform and improve the standard of care are urgently warranted. One of the debated features of COVID‐19 that puzzled clinicians [9, 10] was that acute respiratory syndrome seen in this disease may not be solely explained by lung alterations, raising the possibility that defective oxygen delivery/perfusion might also be implicated.

Anemia, or reduced blood capacity to carry oxygen, may be acute due to blood loss, or associated with a wide range of chronic conditions including inherited and acquired blood disorders, cancer, inflammation, chronic kidney disease, malnutrition, and aging [11, 12]. COVID‐19 severity was found by some [13] but not others [1, 14, 15] to be associated with anemia. Reports from Wuhan suggest a prevalence of anemia in COVID‐19 patients was 15–51% [16], but robust data on the impact of anemia on COVID‐19 disease progression and mortality remains unreported.

Emerging data raise the possibility that red blood cells (RBCs) might be also directly involved in the pathogenesis of the disease. An early report suggested that a protein expressed on RBCs (CD147 or Basigin) that serves as a receptor for plasmodium falciparum [17], might be an additional receptor for SARS‐CoV‐2 [18] [and reviewed in [19]]. These findings led some clinicians to ask whether anemia has an impact on COVID‐19 patient outcome [20, 21, 22, 23].

Here, we asked whether anemia influences COVID‐19 prognosis and patient outcome. The hypothesis tested is that COVID‐19 patients with anemia experience worse clinical course and higher mortality in comparison to COVID‐19 patients with normal RBC parameters.

## 2 METHODS

### 2.1 Patients and outcomes

All patients ≥18 years with an encounter at the Mount Sinai Health System between March 1 and April 1, 2020 who had a COVID‐19 diagnosis that was confirmed by a positive result on a reverse‐transcriptase–polymerase‐chain‐reaction (RT‐PCR) SARS‐CoV‐2 assay of a nasopharyngeal swab specimen were selected (n = 3777) for analysis of patient demographics and comorbidities. Patients from any care setting (ambulatory, emergency department, or hospitalized) were included. Patients who had a complete blood count (CBC, at presentation, n = 2562) were further examined to assess the associations between the primary predictors of interest, (hemoglobin, mean corpuscular volume (MCV), and hematocrit (HCT)) with primary and secondary outcomes (all‐cause mortality and mechanical ventilation). Patient demographics, comorbidity information, laboratory values, mechanical ventilation, and mortality were extracted by physician review of the electronic medical record. Comorbidities were assessed individually, and using the Charlson Comorbidity Index [24, 25], which provides a weighted score of comorbidities, based on the associated increased risk of mortality for each condition. This study was approved by the Program for Protection of Human Subjects (PPHS) of the Icahn School of Medicine at Mount Sinai (IRB # 20–03409).

### 2.2 Classification of laboratory values

Anemia was defined by WHO criteria (hemoglobin levels < 13 g/dL for males and < 12 g/dL for females) [26]. A secondary analysis classified severe, moderate, and mild anemia as hemoglobin < 7 g/dL, 7–9.9 g/dL, and 10–11.9 g/dL (females) or 10–12.9 g/dL (males), respectively. An additional secondary analysis evaluated hemoglobin as a continuous predictor. The normal range for MCV was defined as 80–100 fL.

### 2.3 Statistical analysis

Chi‐squared tests and Wilcoxon rank sum were performed to assess differences in anemia, demographics, and other laboratory values according to mortality. As uniform follow‐up time was not available for each patient, outcomes were modeled using time‐to‐event analysis. A multivariable Cox proportional hazards regression model was performed to assess the independent predictors of mortality utilizing a stepwise selection of patient (age, sex, race, Charlson Comorbidity Index) and clinical (anemia, lymphocyte count, and platelet count) characteristics. An additional stepwise multivariable Cox proportional hazards model was performed to assess the independent predictors of mechanical ventilation status utilizing the same predictors. D‐dimer was intentionally omitted from the regression model given the lack of biologic rationale for inclusion (thereby making confounding unlikely) and to avoid over‐fitting.

A secondary analysis was conducted to explore the sex‐specific optimal hemoglobin levels that best predict mortality. These hemoglobin cut points may help inform the point at which lower hemoglobin levels are of clinical concern. Optimal cutpoints were calculated using the X‐Tile software [27], separately for males and females. The X‐tile software tests each level of hemoglobin as the predictor in a Cox‐proportional hazards model, and calculates the associated log‐rank statistics. The optimal cut‐point is defined at the point with the maximum χ^2^ value, which indicates the greatest difference between the survival distributions of those with and without low hemoglobin levels. The significance of this split was assessed by applying the Miller‐Siegmund *P*‐value correction. A multivariable analysis was rerun with anemia defined according to the optimal cutpoints.

## 3 RESULTS

### 3.1 Patients characteristics

There were 3777 adult patients with an RT‐PCR confirmed diagnosis of COVID‐19 who presented between March 1 and April 1, 2020 at hospitals within the Mount Sinai Health System in New York City, which was the epicenter of the pandemic at the time; 2562 of whom had laboratory data available with a median follow up of 21 (interquartile range [IQR] 11–37) days, censoring patients at time of death. Of patients with laboratory data available, 1,973 (77.0%) were hospitalized. The median age of patients was 59 (IQR 42–71) years and the sample was mostly non‐white or Hispanic (72.6%) (Table 1). Forty‐five percent were female. Fifty‐four percent of patients had no reported comorbidities. Mechanical ventilation was required for 14.1% (n = 531) of the patients and 16.7% (n = 629) of the patients died at time of last follow up (Table 1).

**TABLE 1:** Description of the study sample by availability of laboratory data (n = 3777)

Variable	Labs (n = 2563, 67.9%) N (%)	No Labs (n = 1214, 32.1%) N (%)	*P*‐value
Sex			
Male	1459 (56.9)	610 (50.3)	.0001
Female	1104 (43.1)	604 (49.8)	
Race^‡^			
Non‐Hispanic white	632 (24.7)	381 (33.4)	<.0001
Non‐white, Hispanic	1931 (75.3)	760 (66.6)	
Age (years)^‡^			
< 52	628 (24.5)	735 (64.4)	<.0001
52 ‐ 64	605 (23.6)	253 (22.2)	
64 ‐ 75	707 (27.6)	124 (10.9)	
> 75	622 (24.3)	29 (2.5)	
Comorbidities			
Myocardial Infarction	134 (5.2)	17 (1.4)	<.0001
Congestive Heart Failure	275 (10.7)	21 (1.7)	<.0001
Peripheral Vascular Disease	135 (5.3)	5 (0.4)	<.0001
Stroke or TIA*	205 (8.0)	8 (0.7)	<.0001
Dementia	196 (7.7)	7 (0.6)	<.0001
COPD^†^	160 (6.2)	8 (0.7)	<.0001
Connective Tissue Disease	59 (2.3)	13 (1.1)	.0098
Peptic Ulcer Disease	23 (0.9)	9 (0.7)	.6251
Liver Disease	57 (2.2)	5 (0.4)	<.0001
Diabetes	516 (20.1)	103 (8.5)	<.0001
Diabetes with end organ damage	417 (16.3)	22 (1.8)	<.0001
Chronic Kidney Disease Stage III	331 (12.9)	22 (1.8)	<.0001
Solid Cancers	216 (8.4)	23 (2.7)	<.0001
Leukemia/Lymphoma	66 (2.6)	9 (0.7)	.0002
AIDS	11 (0.4)	4 (0.3)	.6491
Hemiplagia	24 (0.9)	2 (0.2)	.0074
Charlson Comorbidity Score			
Mean (SD)	1.7 (2.1)	0.4 (1.0)	<.0001
BMI (kg/m^2^)^#^			
≤ 30	1523 (59.4)	665 (54.8)	<.0001
> 30	879 (34.3)	221 (18.2)	
Mechanical Ventilation			
No	2045 (80.0)	1201 (98.9)	<.0001
Yes	518 (20.2)	13 (1.1)	
Anemia^§^ (< 12 g/dL Hemoglobin, females; < 13 g/dL, males)			
Yes	793 (30.9)		
No	1770 (69.1)		
Absolute Lymphocyte Count^$^ (# x 10^9^ / L)			
< 1	848 (44.4)		
≥1	1066 (55.7)		
Platelet count^¶^ (# x 10^9^ / L)			
< 150	438 (22.5)		
≥150	1510 (77.5)		

* Transient Ischemic Attack.

^†^ Chronic Obstructive Pulmonary Disease.

^‡^ Race was missing for 73 patients and age was missing for 74 patients.

$ BMI information missing for 489 patients.

^§^ Laboratory measurements reported on 2,563 patients, 1 patient dropped in subsequent analyses due to missing age.

^¶^ 2,558 patients with valid platelet measures.

^#^ 2,507 patients with valid lymphocyte measures.

Mortality was significantly associated with increasing age (*P* < .0001), male sex (*P* < .0001), a higher Charlson comorbidity score (*P* < .0001), and mechanical ventilation (*P* < .0001) (Table 2). In this cohort, there was no statistically significant difference in mortality by race (Table 2, *P* = .45).

**TABLE 2:** Description of study sample according to mortality status (n = 3777)

Variable	Alive (n = 3148, 83.3%) N (%)	Deceased (n = 629, 16.7%) N (%)	*P*‐value
Sex			
Male	1680 (53.4)	389 (61.8)	<.0001
Female	1468 (46.6)	240 (38.2)	
Race^‡^			
Non‐Hispanic white	835 (27.1)	178 (28.6)	.4529
Non‐white, Hispanic	2246 (72.9)	445 (71.4)	
Age (years)^‡^			
< 52	1325 (43.0)	38 (6.1)	<.0001
52 ‐ 64	751 (24.4)	107 (17.2)	
64 ‐ 75	628 (20.4)	203 (32.6)	
> 75	377 (12.2)	274 (44.1)	
Comorbidities			
Myocardial Infarction	99 (3.1)	52 (8.3)	<.0001
Congestive Heart Failure	178 (5.7)	118 (18.8)	<.0001
Peripheral Vascular Disease	92 (2.9)	48 (7.6)	<.0001
Stroke or TIA*	135 (4.3)	78 (12.4)	<.0001
Dementia	102 (3.2)	101 (16.1)	<.0001
COPD^†^	108 (3.4)	60 (9.5)	<.0001
Connective Tissue Disease	61 (1.9)	11 (1.8)	.7517
Diabetes	492 (15.6)	127 (20.2)	.0048
Diabetes with end organ damage	264 (8.4)	175 (27.8)	<.0001
Chronic Kidney Disease Stage III	232 (7.4)	121 (19.2)	<.0001
Localized Solid Tumor	151 (4.8)	46 (7.3)	.0096
BMI (kg/m^2^)^#^			
BMI ≤ 30	1795 (63.0)	393 (64.6)	.4796
BMI > 30	888 (37.0)	212 (35.4)	
Charlson Comorbidity Score			
Mean (SD)	1.01 (1.7)	2.58 (2.3)	<.0001
Anemia^§^ (< 12 g/dL Hemoglobin, females; < 13 g/dL, males)			
Yes	534 (27.4)	258 (42.4)	<.0001
No	1418 (72.6)	352 (57.6)	
Absolute Lymphocyte Count^#^ (# x 10^9^ / L)			
< 1	848 (44.4)	312 (52.6)	.0004
≥1	1066 (55.7)	281 (47.4)	
Platelet count^¶^ (# x 10^9^ / L)			
< 150	438 (22.5)	167 (27.4)	.01
≥150	1510 (77.5)	443 (72.6)	
Mechanical Ventilation			
No	2999 (95.3)	247 (39.3)	<.0001
Yes	149 (4.7)	382 (30.7)	

* Transient Ischemic Attack.

^†^ Chronic Obstructive Pulmonary Disease.

^‡^ Race and age was missing for 73 patients and age was missing for 74 patients.

^§^ Laboratory measurements reported on 2,562 patients.

^¶^ 2,558 patients with valid platelet measures.

^#^ 2,507 patients with valid lymphocyte measures. .

### 3.2 Anemia in patients with COVID‐19

Further analyses were performed on 2562 (67.9%) of the patients with laboratory measurements of hemoglobin, RDW, and MCV. The median time from COVID‐19 diagnosis to the collection of laboratory parameters was 0 (IQR 0–1) days. Seven hundred ninety‐two (30.9%) patients met the clinical criteria for anemia; 311 (12.1%) patients had MCV values outside a normal range, while 502 (19.6%) patients had RDW values above normal (Table 3). In addition, 605 (23.6%) patients had thrombocytopenia and 1,159 (46.3%) had lymphopenia.

**TABLE 3:** Description of patients according to hemoglobin levels (n = 2562)

		Anemia	*P*‐value
		No (n = 1770, 69.3%) N(%)	Yes (n = 792, 30.9%) N(%)
Sex			
Male	1007 (56.9)	451 (57.0)	.9600
Female	763 (43.1)	341 (43.0)	
Race			
Non‐Hispanic white	443 (25.0)	189 (23.9)	.5166
Non‐white, Hispanic	1327 (75.0)	603 (76.1)	
Age (years)^⋀^			
< 52	482 (27.3)	146 (1845)	<.0001
52 ‐64	449 (25.4)	156 (19.7)	
64 – 75	483 (27.3)	224 (28.3)	
> 75	356 (20.1)	266 (33.6)	
Charlson Comorbidity Score			
Mean (SD)	1.17 (1.7)	2.92 (2.4)	<.0001
Hematocrit (%) mean (SD)			
Males	44.4 (3.6)	33.8 (4.9)	<.0001
Females	41.0 (3.4)	32.2 (4.2)	<.0001
Red blood cell distribution width (%)			
> 15	151 (8.5)	350 (44.3)	<.0001
≤ 15	1619 (91.5)	442 (55.7)	
Mean corpuscular volume (fL)			
< 80	74 (4.2)	114 (14.4)	<.0001
80–100	1640 (92.7)	611 (77.2)	
> 100	56 (3.2)	67 (8.5)	
Absolute Lymphocyte Count"/> (# x 10^9^ / L)			
< 1	778 (44.5)	381 (50.4)	.007
≥1	970 (55.5)	375 (49.6)	
Platelet count*(# x 10^9^ / L)			
< 150	394 (22.3)	211 (26.7)	.01
≥150	1374 (77.7)	578 (73.3)	
Mechanical Ventilation			
No	1430 (80.8)	615 (77.6)	.067
Yes	340 (19.2)	177 (22.4)	

* 2557 patients with valid platelet and hemoglobin measures.

^†^ 2504 patients with valid lymphocyte and hemoglobin measures. .

As anticipated, anemia was statistically significantly associated with increasing age (*P* < .0001), and higher Charlson comorbid index scores (*P* < .0001). Also as expected, patients with anemia were more likely to have a lower HCT in both males and females (*P* < .0001), RDW > 15% (*P* < .0001), and MCV outside the range of 80–100 fL (*P* < .0001; Table 3). As expected, anemia was also significantly associated with thrombocytopenia (*P* = .01) and lymphopenia (*P* = .007)

The relationship between anemia and outcomes was further evaluated by applying a multivariable model. After stepwise selection, the model for mortality included age, sex, and Charlson comorbidity index. We found anemia to be independently significantly associated with a higher risk of mortality (HR_adj_: 1.26, 95% Confidence Interval [CI]: 1.06–1.51; Table 4). However, anemia was not significantly independently associated with mechanical ventilation after adjustment for age, sex, race, and lymphocyte count (HR_adj_: 1.11, 95% CI: 0.92‐1.34; Table 5).

**TABLE 4:** Independent predictors of mortality among COVID‐19 positive patients

	n = 2497"/>
	HR^‡^_adj_ (95% CI)
Anemia Yes vs No*	1.26 (1.06 ‐ 1.51)
Female vs Male	0.67 (0.60 ‐ 0.84)
Age (years) < 52	1.0 (ref)
52 ‐ 63	2.39 (1.65 – 3.48)
64–75	3.85 (2.71 – 5.47)
> 75	6.66 (4.70 – 9.44)
Charlson Comorbidity Index (continuous)	1.06 (1.02 ‐ 1.10)

* (< 12 g/dL Hemoglobin, females; < 13 g/dL, males).

^†^ 65 patients excluded from analysis for missing data.

^‡^ Adjusted for all variables listed.

**TABLE 5:** Predictors of mechanical ventilation among COVID‐19 positive patients

	n = 2,485"/>
	HR^‡^_adj_ (95% CI)
Anemia: Yes vs No*	1.11 (0.92‐1.34)
Female vs Male	0.79 (0.65 ‐ 0.94)
Non‐Hispanic white vs Non‐white, Hispanic	0.81 (0.65 – 1.00)
Age (years) < 52	
52 ‐63	1.69 (1.27 – 2.62)
64–75	2.28 (1.74 – 2.99)
> 75	1.59 (1.18 ‐ 2.15)
Absolute Lymphocyte Count (# x 10^9^ / L)	
≥1	1.0 (ref)
< 1	1.24 (1.03‐1.48)

* (< 12 g/dL Hemoglobin, females; < 13 g/dL, males).

^†^ 77 patients excluded from analysis for missing data.

^‡^ Adjusted for all variables listed.

Secondary analyses of hemoglobin as a continuous variable, or categorized according to severity showed similar results. In a multivariable model adjusted for age, sex, race, and Charlson comorbidity index, there was a statistically significant increase in mortality (HR_adj_ 1.05; [CI]: 1.01‐1.09) with decreasing hemoglobin levels. However, decreasing hemoglobin levels were not significantly independently associated with mechanical ventilation (HR_adj_ 1.02; [CI]: 0.98–1.07). Similar results were obtained across severity categories of anemia, although the highest category contained very few cases.

### 3.3 Optimal hemoglobin cutpoints to predict outcomes

Next, we aimed to determine the hemoglobin level that is maximally predictive of mortality, using the X‐Tile software. For males, the optimal hemoglobin cutpoint was defined at 11.5 g/dL (Miller‐Seigmund *P* < .0001) (Figure S1a). In females, the optimal cutpoint was defined at 11.8 g/dL (Miller‐Seigmund *P* = .0787) (Figure S1b). When the optimal hemoglobin cut points were applied to the model instead of the WHO definition of anemia, a stronger association (HR_adj_ 1.40, 95% CI: 1.17‐1.69), was observed between hemoglobin and mortality after adjustment for confounders.

## 4 DISCUSSION

The results of this study indicate previously unrecognized association between decreasing hemoglobin levels and increased risk of mortality in COVID‐19 patients, independent of age, sex, comorbidities, and other hematologic parameters including lymphocyte and platelet count. While considered among other known risk factors, anemia may serve as an early indicator of poor outcomes in patients with COVID‐19. These results may provide a rationale for including anemia in algorithms assessing risk prediction in COVID‐19 mortality and severity.

Although modulations of red cell distribution width (RDW) are thought to predict disease severity and worse outcomes [28], RBC indices are often underappreciated especially in patients with only mild anemia. We originally hypothesized that severe anemia would be predictive of COVID‐19 severity and mortality. Surprisingly, our analysis found that the ideal hemoglobin cutoffs for considering anemia as a significant contributor to mortality was 11.5 g/dL in males and 11.8 g/dL in females. The maximally predictive hemoglobin for mortality in male patients was only just below the WHO criteria for anemia, interestingly, suggesting that even mild compromises in hemoglobin reserve can be prognostically detrimental in COVID‐19. Recently published evidence has also suggested than an elevation in RDW may be prognostic in hospitalized COVID‐19 patients [29]. Interestingly, in our cohort we observed an association between RDW and mortality only in non‐anemic patients (manuscript under review).

The impact of anemia observed in COVID‐19 in this study has precedent. Anemia is known to be a poor prognostic marker in several other respiratory diseases. In chronic obstructive pulmonary disease (COPD), anemia is a well validated independent predictor of mortality [30, 31]. Similarly, in congestive heart failure, a hemoglobin < 13 g/dL is independently associated with an increased risk of hospitalization and death [32]. In intubated patients in the intensive care unit, hemoglobin < 10 g/dL is associated with a five‐fold increase in the risk of unsuccessful extubation [33]. The impact of anemia on COVID‐19 outcomes may in part be a consequence of the highly inflammatory state associated with COVID‐19. Anemia may be an independent risk factor for poor outcome for reasons that are not yet evident and require further investigations. In this context, the relationship between erythroid cell production, iron metabolism, and inflammatory signaling may be relevant [34, 35, 36, 37, 38, 39]. However, it is important to note that acute anemia in the critically ill is almost always caused by acute bleeding and hemolysis [40]. However, elevation of circulating IL‐6 levels on hepcidin and its modulation of iron availability and red blood cell production may limit the compensatory erythropoietic response in COVID‐19 patients [41, 42, 43, 44].

Interestingly, the association between anemia and mechanical ventilation on multivariable analysis was not significant. The decision to intubate is clinically made, with variation in practice among clinicians. Therefore, mechanical ventilation is subject to a degree of bias. As an example, patients who are greater than 75 had a lower OR of intubation as compared with patients 64–75 (Table 5).

The current study has limitations that are important to recognize. This study was unable to robustly explore prior history of anemia in our patient cohort, nor was it able to investigate baseline hematological indices in the patient population specifically in patients with hemoglobinopathies. Our study also lacked information about symptom onset timing, therefore we were unable to determine whether a patient's anemia was pre‐existing or a result of a worsening COVID‐19 infection. Our study was also limited by inability to assess the degree to which hemolysis may be contributing to baseline anemia. LDH was universally elevated in our patient cohort and haptoglobin levels were rarely obtained in this retrospective dataset. Patients with available lab data represent those with a higher risk burden, and likely a more serious infection in need of clinical care and intervention, while those without lab data may have been tested in outpatient environments, including rapid testing centers, or may not have reported clinical manifestation of symptoms for COVID‐19. In addition, patients presenting to care had variable severity of acute disease and inflammation, which we were unable to evaluate and might be an important confounder as the impact of acute inflammation on anemia remains unclear. We were also unable to assess a broad set of socio‐economic predictors of disease outcomes such as patient income or living conditions, which have been increasingly underlined as highly important in determining outcomes in the New York City outbreak.

Strengths of our study include a large population of COVID‐19 patients with common hematological parameters collected at multiple sites in New York City. Our study was also able to measure a complete Charlson comorbidity index, which is a powerful and well‐validated measure of overall mortality outcomes in the general population [24, 25].

These results have potential clinical implications in terms of risk stratification and early implementation of treatment strategies. These may include reducing the inflammatory consequences of COVID‐19 respiratory borne viral infection. The heightened inflammatory state drives pulmonary compromise and multi‐organ failure and likely intersects with the clotting cascade [45, 46]. Agents that are currently in clinical evaluation in targeting the pathological inflammatory cytokine production include inhibitors of Janus kinase (JAK) 2 and Bruton tyrosine kinase (BTK) as well as selective cytokine antagonists [47, 48]. In addition, dexamethasone, a potent anti‐inflammatory, has been shown to decrease mortality in COVID‐19 patients who are hospitalized and requiring respiratory support [49].

Identification of a patient at higher risk for complications of COVID‐19 would allow for a risk‐adapted approach with early intervention employing such therapeutics that may mitigate the inflammation‐mediated organ damage and thrombo‐inflammation and coagulopathy that significantly contributes to mortality. As prospective studies evaluate various rational therapies for COVID‐19 often re‐purposed from other approved indications, it will be important to stratify for baseline clinical variables such as anemia, as shown in this study, in order to determine whether the intervention has specific benefit in high‐risk subsets. Steroids have been shown to have a positive effect on COVID‐19 outcome and are now increasingly incorporated into institutional treatment guidelines [49].

In summary, anemia is a critical previously unrecognized independent predictor of poor outcomes in patients with COVID‐19. These readily available biomarkers should be considered by clinicians when risk‐stratifying a patient with COVID‐19 and may eventually identify a subset of patients that require early specific therapeutic intervention. The underlying pathological relationship of mild anemia with poor outcomes may at least in a subset of patients be linked to inflammation, thereby, informing a targeted treatment approach, however, further investigation is required.

## AUTHOR CONTRIBUTIONS

DT, JR, JM, ET, and SG conceived and designed the study. JR, NA, and WLC performed the statistical analyses, DT, JR, NA, and ET analyzed the data. All authors participated in writing and editing the manuscript.

## CONFLICT OF INTEREST

The authors declare no relevant conflict of interest

## Supporting information



Supplementary Figure 1‐ Distribution of haemoglobin levels and Kaplan‐Meier survival estimates of mortality for those with and without low haemoglobin for (a) males and (b) females. For males, the optimal cutpoint was 11.5 g/dL; for females the optimal cutpoint was 11.8 g/dL.

## Data Availability

The data that support the findings of this study are available from the corresponding author upon reasonable request.
