# Supplementary figures and images for: Mild anemia as a single independent predictor of mortality in patients with COVID‐19
Source: EJHaem. 2021 May 6;2(3):319–26. doi: 10.1002/jha2.167 (PMC8242891; doi:10.1002/jha2.167)

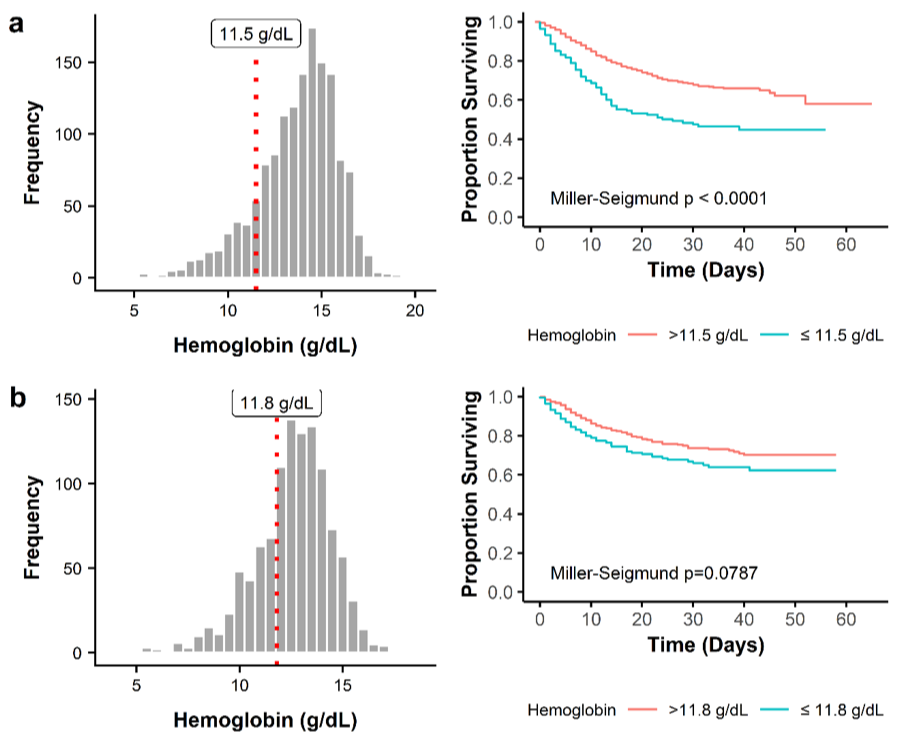

Supplement: Supplementary file 1 — Supplementary Figure 1‐ Distribution of haemoglobin levels and Kaplan‐Meier survival estimates of mortality for those with and without low haemoglobin for (a) males and (b) females. For males, the optimal cutpoint was 11.5 g/dL; for females the optimal cutpoint was 11.8 g/dL. [file JHA2-2-319-s001.png]
